# Value of routine blood tests for prediction of mortality risk in hip fracture patients

**DOI:** 10.3109/17453674.2011.652883

**Published:** 2012-02-08

**Authors:** Mathias Mosfeldt, Ole B Pedersen, Troels Riis, Henning O Worm, Susanne van Mark, Henrik L Jørgensen, Benn R Duus, Jes B Lauritzen

**Affiliations:** ^1^Departments of Orthopedic Surgery; ^2^Clinical Biochemistry, Bispebjerg University Hopital, Copenhagen; ^3^Tissue Typing Laboratory, Department of Clinical Immunology, Copenhagen University Hospital, Copenhagen, Denmark

## Abstract

**Background:**

There is a 5- to 8-fold increased risk of mortality during the first 3 months after a hip fracture. Several risk factors are known. We studied the predictive value (for mortality) of routine blood tests taken on admission.

**Methods:**

792 hip fracture patients were included prospectively; blood tests were taken on admission. Follow-up data on mortality were obtained from the civil registration system. Patients were divided into 2 groups based on whether they had survived at least 90 days after the hip fracture. To estimate which laboratory tests could be used to predict outcome, we used receiver operation characteristic (ROC) curves.

**Results:**

3-month mortality could be predicted with 69% accuracy from the level of plasma creatinine in standard admission blood tests. The mortality in patients with elevated levels of creatinine was almost 3-fold that of the patients with normal creatinine. Mortality was also associated with age, low blood hemoglobin, high plasma potassium, and low plasma albumin levels.

**Interpretation:**

Our findings could be of use in identifying patients who might benefit from increased attention perioperatively.

With the increasingly large proportion of elderly individuals in our populations, the hip fracture incidence curve is shifting to the right and hip fracture patients are becoming a more heterogeneous group (Bergström et al. 2009). Almost all excess mortality occurs within the first 3 months after surgery (Petersen et al. 2006). A large meta-analysis has found a 5- to 8-fold increased risk of mortality during this period (Haentjens et al. 2010). Several factors that may be predictive of death have been identified (Petersen et al. 2006). We studied the predictive value (for mortality) of routine blood tests.

## Patients and methods

792 hip fracture patients aged 60 or older who were admitted to the Department of Orthopedics, Bispebjerg University Hospital, Denmark from October 2008 through July 2010 were included prospectively. 67 patients aged less than 60 were excluded. Follow-up data on mortality were obtained from the civil registration system on August 1, 2010. Patients were included in the study within the first 3 days after admission.

Because of various logistical problems, not all blood tests were done for some patients in the study (Table 1). The patients were divided into 2 groups based on whether they survived for at least 90 days after the hip fracture.

**Table 1.** Characteristics of the cohort and blood sample values at the time of admission

	Hip fracture patients			
	Number of patients	Survived study period	Died during study period	p-value
Number	792	560	232	
Age, mean (SD)				
Women	591	83 (9)	87 (8)	< 0.001
Men	201	78 (9)	83 (9)	< 0.001
Sex, n (%)				
Women	591	425 (76%)	166 (72%)	
Men	201	135 (24%)	66 (28%)	0.2
Blood hemoglobin (mmol/L), mean (SD).				
Reference interval: women: 7.3–9.5 mmol/L, men: 8.5–10.3 mmol/L				
Women	590	7.5 (1.1)	7.3 (1.0)	0.02
Men	201	8.0 (1.2)	7.2 (1.2)	< 0.001
Plasma creatinine (μmol/L), median (range).				
Ref. int.: women: 50–90 μmol/L, men: 60–105 μmol/L				
Women	585	68 (33–445)	82 (36–615)	< 0.001
Men	200	82 (39–419)	106 (50–699)	< 0.001
Plasma sodium (mmol/L), mean (SD).				
Ref. int.: 137–144 mmol/L				
Women	587	137 (4.1)	137 (5.1)	0.4
Men	201	138 (4.3)	138 (4.6)	0.5
Plasma potassium (mmol/L), mean (SD).				
Ref. int.: 3.5–4.6 mmol/L				
Women	587	3.8 (0.49)	4.0 (0.67)	0.002
Men	201	4.0 (0.49)	4.1 (0.59)	0.2
Plasma glucose (mmol/L), mean (SD).				
Ref. int.: 4.2–7.2 mmol/L				
Women	556	6.8 (2.1)	6.5 (1.7)	0.1
Men	185	6.6 (2.3)	6.8 (2.1)	0.5
Plasma albumin (g/L), mean (SD).				
Ref. int.: 34–45 g/L				
Women	515	39 (4.4)	37 (5.4)	< 0.001
Men	174	39 (4.7)	36 (5.6)	0.003
Plasma CRP (mg/L), median (range).				
Ref. int.: < 10 mg/L				
Women	458	67 (82)	71 (73)	0.05
Men	170	66 (70)	81 (86)	0.2
Leukocyte count (× 10^9^/L), mean (SD).				
Ref. int.: 3.5–8.8 × 10^9^/L				
Women	590	11.0 (4.0)	10.8 (4.1)	0.7
Men	201	11.5 (5.6)	10.5 (4.3)	0.1
Platelet count (× 10^9^/L), mean (SD).				
Ref. int.: 145–390 × 10^9^/L				
Women	590	249 (86)	270 (80)	0.007
Men	201	229 (79)	240 (90)	0.4

Blood was analyzed for hematology and chemistry using Sysmex (Sysmex Corporation, Kobe City, Japan) and Vitros 5.1 FS (Ortho-Clinical Diagnostics, Pisataway, NJ) respectively, using standardized routine laboratory methods.

The study was approved by the local ethics committee and by the Danish data protection agency, and it was carried out in line with the Helsinki Declaration.

### Statistics

Continuous variables are presented as mean (SD) or median (range). We used student's t-test for continuous, normally distributed variables, Mann-Whitney 2-sample statistics for skewed distributions, and Chi-squared test to compare categorical variables. To estimate which laboratory tests could be used to predict outcome, we used receiver operation characteristic (ROC) curves and calculated the area under the curve (AUC) for the different tests. Cutoff values for the different laboratory tests were predicted using ROC curves or based on the test performance in a normal cohort. Univariate and multivariate logistic regression analyses were used to examine the relationship between the various risk factors and outcome. Values of p < 0.05 were considered statistically significant. Data were analyzed using STATA software.

## Results

**Figure 1.**
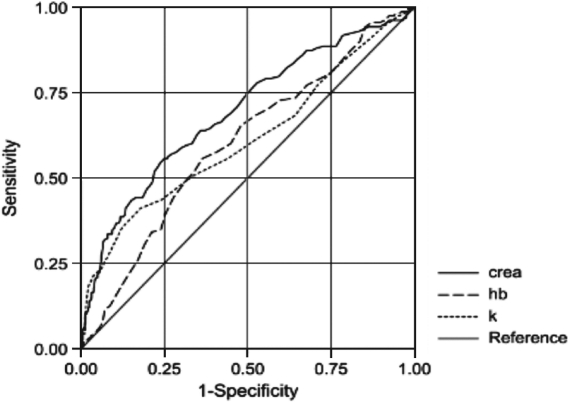
ROC curves for prediction of mortality in relation to laboratory values at admission. The curves represent plasma creatinine (crea, solid line), inverse blood hemoglobin (hb, dashed line), and plasma potassium (K, dotted line). Hemoglobin values were converted because the association with death followed decreasing values.

232 of 792 patients (29%) died during the study period (Table 1), at median 44 (1–582) days. 57 died between 1 and 66 days, while still in hospital. 106 died after leaving hospital but within 3 months, and 69 died some time after 3 months. Based on the ROC curves (Figure 1), the cutoff values were set at 7 mmol/L in both sexes for hemoglobin and above 50 mg/L for C-reactive protein (CRP). The ROC curves for most other laboratory tests did not indicate any optimal cutoff value (the number of correctly classified patients was the same for a large variety of values) (Table 2). We therefore decided to use the upper or lower limits of normal laboratory reference intervals as cutoff values.

**Table 2.** Area under the curve (AUC) for the routine laboratory tests included in the study

	Area under ROC **^a^**	SE **^b^**	95% CI
Plasma creatinine	0.69	0.02	0.64–0.74
Plasma potassium	0.61	0.02	0.55–0.66
Blood hemoglobin	0.60	0.02	0.55–0.65
Plasma albumin	0.59	0.02	0.53–0.64
Blood platelet count	0.58	0.02	0.53–0.63
Plasma CRP	0.55	0.02	0.50–0.60
Blood glucose	0.52	0.02	0.47–0.57
Blood leukocyte count	0.52	0.02	0.47–0.57
Plasma sodium	0.51	0.02	0.46–0.56

**^a^** Receiver operating curve

**^b^** standard error

**Figure 2.**
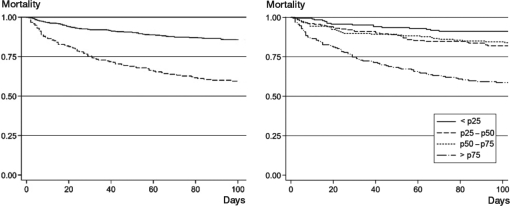
Kaplan Meier plot: mortality in relation to creatinine levels at admission. 3-month mortality after hip fracture. The plot on the left is divided into groups with elevated plasma creatinine (dotted line) and normal plasma creatinine (solid line). Plasma creatinine was elevated if above 105 μmol/L in males and 90 μmol/L in females. The plot on the right is divided into quartiles of plasma creatinine.

In the univariate analysis (Table 3), high age, high plasma creatinine (Figure 2), low plasma albumin, high plasma potassium, and low blood hemoglobin led to increased mortality. Patient mortality was not related to gender, blood platelet count, or CRP. After adjustment in the multivariate logistic regression model, age, plasma creatinine, plasma potassium, plasma albumin remained as risk factors.

**Table 3.** Unadjusted and adjusted risk factors influencing mortality after hip fracture

Risk factors	Died	Survivors	Unadjusted OR	p-value	Adjusted OR	p-value
	(%)	(%)	(95% CI)		(95% CI)	
Sex (male)	29	24	1.26 (0.85–1.85)	0.3	1.33 (0.86–2.06)	0.2
Age > 80 years	85	64	3.21 (2.03–5.09)	< 0.001	2.92 (1.78–4.81)	< 0.001
Creatinine (> 90 and >105 μmol/L in women and men, respectively)	52	21	4.19 (2.91–6.03)	< 0.001	2.84 (1.90–4.25)	< 0.001
Potassium (> 4.5 mmol/L)	22	4	6.10 (3.59–10.4)	< 0.001	3.64 (2.01–6.61)	< 0.001
Hemoglobin(< 7 mmol/L)	40	25	1.92 (1.34–2.77)	< 0.001	1.13 (0.75–1.71)	0.6
Albumin (< 34 g/L)	8	3	3.04 (1.48–6.27)	0.002	3.08 (1.33–7.15)	0.009
Platelet count (> 390 × 10^9^/L)	9	5	1.78 (0.95–3.32)	0.07	1.43 (0.69–2.94)	0.3
CRP (> 50 mg/L)	59	56	1.16 (0.82–1.65)	0.4	1.11 (0.75–1.62)	0.6

## Discussion

Previous studies on mortality after hip fracture have found an association with plasma creatinine levels (Lewis et al. 2006, Singh et al. 2008, Björkelund et al. 2009, Bennet et al. 2010, Ho et al. 2010), which is what we found in the present study. Of these previous studies, the most comprehensive was a prospective observational study of 2,963 consecutive patients by Lewis et al. (2006). They found that serum urea was an independent predictor of mortality at 30 and 90 days, and also at 1 and 2 years. Furthermore, mortality was also higher in patients admitted with raised or low serum sodium, raised serum potassium, and raised serum creatinine. A prospective cohort study of 436 patients in Sweden found that in bivariate analysis, a creatinine concentration of more than 100 umol/L on admission was a risk factor for poor 4-month survival. Using multiple logistic regression analysis, creatinine was not found to be an independent risk factor (Björkelund et al. 2009).

In both of the above-mentioned studies, the cutoff value for elevated creatinine was set differently than in our study, and it appears that the same value was used for both sexes. In our study, the cutoff level for elevated serum creatinine was set at 105 μmol/L for men and 90 μmol/L for women, as these are the upper limits of 95% CI in normal Scandinavian subjects (Felding et al. 2004).

A recent Norwegian study on 364 hip fracture patients found that the use of diuretics, followed by the presence of congestive heart disease on admission, were the strongest predictors of 1-year mortality (Juliebø et al. 2010). These findings suggest that the increased mortality in patients with elevated creatinine may be due to congestive heart disease that is severe or poorly regulated. The added strain of hospitalization and surgery may lead to the increased mortality. Concerning serum albumin, several previous studies have found it to be associated with mortality after hip fractures (Koval et al. 1999, Pioli et al. 2006, O'Daly et al. 2010, Pimlott et al. 2011, Oztürk et al. 2010).

A recent retrospective study of 200 patients (O'Daly et al. 2010) suggested that serum albumin at admission and total lymphocyte count could serve as markers of malnutrition and could be used to predict clinical outcome after hip fractures. However, only levels of albumin at admission and age were found to be independent prognostic factors for 1-year mortality by Cox regression analysis, with hazard ratios of 0.93 (95% CI: 0.89–0.98; p = 0.007) and 1.04 (95% CI: 1.007–1.07; p = 0.02) respectively. In the same study, patients with low albumin at admission had an odds ratio of 4.0 (95% CI: 1.3–12.2; p = 0.02) for dying within 3 months of the fracture (as compared to patients with normal albumin levels). In a similar study (Koval et al. 1999), patients were regarded as being malnourished based on total lymphocyte count and albumin levels at admission. Patients with reduced values for both parameters on admission were 3.5 times more likely to die within 1 year of surgery (p < 0.01).

A recent study from Canada of 583 hip fracture patients found an association between low serum albumin levels at admission and in-hospital mortality (Pimlott et al. 2010). After multivariate adjustment, an association between low serum albumin and mortality was found to be statistically significant with an adjusted OR of 2.4 (95% CI: 1.2–5.1; p < 0.05). Almost half of the hip fracture patients had low serum albumin at the time of admission and it was independently associated with 2.5-fold greater odds of short-term mortality.

The association between mortality after hip fracture and reduced hemoglobin levels at admission has also been reported in previous studies (Gruson et al. 2002, Halm et al. 2004, Bhaskar and Parker 2011). In a American study of 548 hip fracture patients (Halm et al. 2004), 60-day mortality after hip fracture was associated with admission levels of hemoglobin, giving an adjusted odds ratio of 0.69 (95% CI: 0.49–0.95; p < 0.05). The authors theorized that the degree of anemia is a marker of underlying comorbid illness burden and physiological reserve. A study on 395 patients found that patients with anemia on admission were more likely to die within 6 or 12 months of hip fracture surgery (Gruson et al. 2002). The severity of anemia was associated with an increased risk of mortality. In the same study, anemia on admission was not predictive of 3-month mortality, but was predictive of mortality at 6 and 12 months after hip fracture.

The strength of our study is that the mortality data has been validated. However, we have no information on the cause of death. It is uncertain whether preoperative correction of elevated serum creatinine levels would be beneficiary, but we are currently planning studies to investigate this.
